# Supplementary material for: Spatial control of myosin regulatory light chain phosphorylation modulates cardiac thick filament mechanosensing
Source: Proc Natl Acad Sci U S A. 2026 Feb 5;123(6):e2520471123. doi: 10.1073/pnas.2520471123 (PMC12891028; doi:10.1073/pnas.2520471123)
Supplement: Supplementary file 1 — Appendix 01 (PDF) [file pnas.2520471123.sapp.pdf]

Supporting Information for

Spatial control of myosin regulatory light chain phosphorylation modulates cardiac thick filament mechano-sensing.

Caterina Squarci, Daniel Koch, Paul Aanaya, Kenneth S. Campbell and Thomas Kampourakis

Thomas Kampourakis
Email: thomas.kampourakis@uky.edu

This PDF file includes:

Figures S1 to S6

Figures

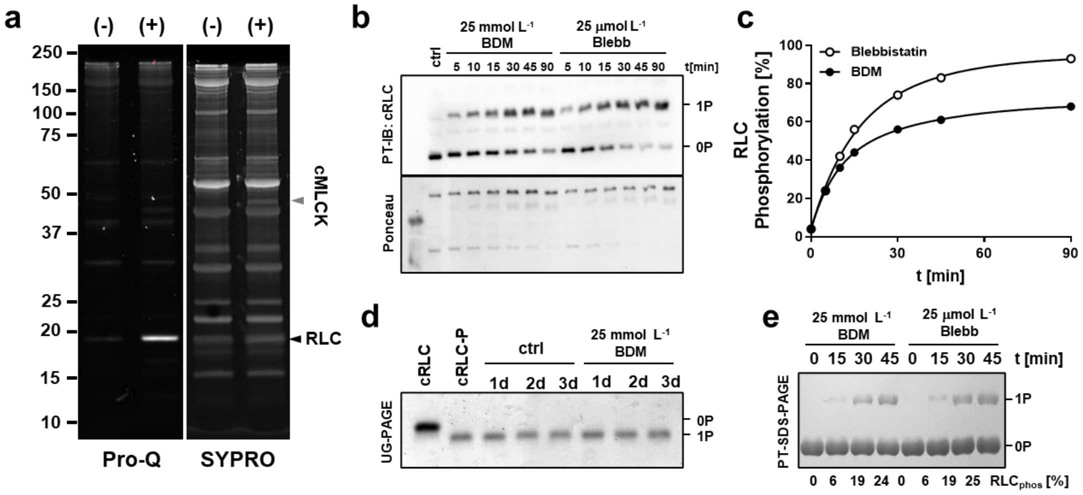

Fig. S1. (a) Pro-Q Diamond and SYPRO Ruby staining of SDS-PAGE separating rat ventricular myofibrillar proteins before (-) and after cMLCK treatment (+). (b) PhostagTM-Western-blot of the time-dependent phosphorylation of RLC in ventricular myofibrils in the presence of either butadiene, 2,3-monoxime (BDM) or blebbistatin (Blebb). (c) Time course of RLC phosphorylation shown in (b). (d) Isolated recombinant phosphorylated RLC was incubated in the absence (ctrl) or in the presence of 25 mmol L-1 BDM for up to three days at 25ºC, and the RLC phosphorylation level determined by urea-glycerol PAGE (UG-PAGE). (e) Effect of BDM or Blebb on the phosphorylation of isolated RLC by cMLCK analyzed by PhostagTM-SDS-PAGE. The relative amount of phosphorylated RLC is indicated below the gel.

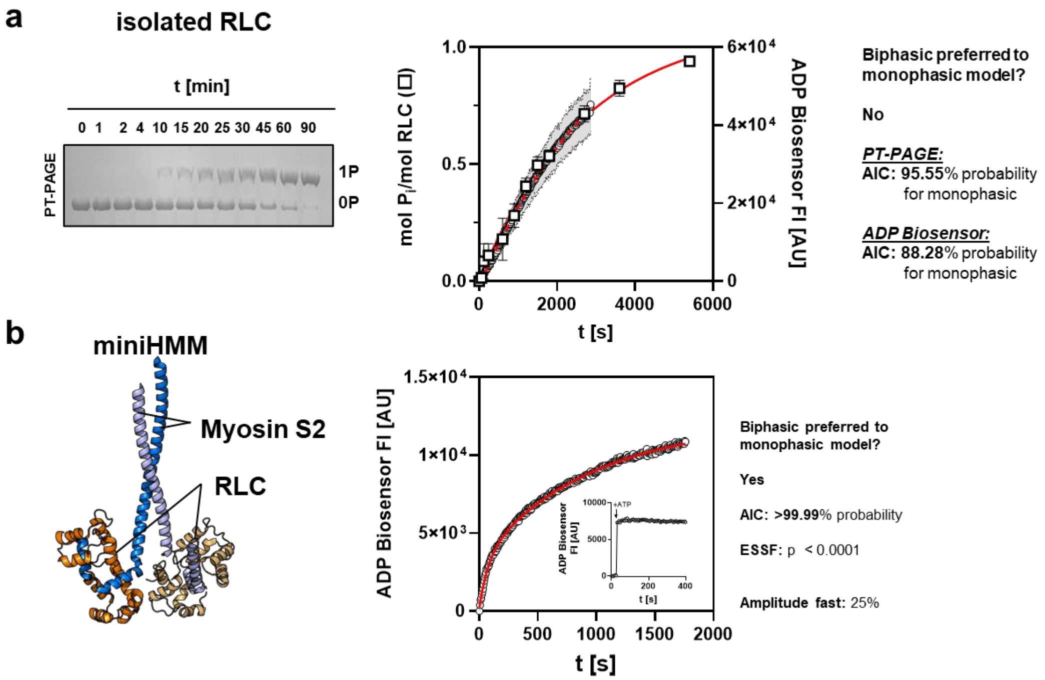

Fig. S2. (a) Time-dependent phosphorylation of isolated RLC by cMLCK analyzed by Phostag™-SDS-PAGE (white squares) and ADP-biosensor assay (white circles). Red continuous line denotes fit to a mono-exponential function. (b) Time-dependent phosphorylation of isolated miniHMM by cMLCK analyzed by ADP-biosensor assay (white circles). Red continuous line denotes fit to a bi-exponential function. Inset shows control experiments in the absence of miniHMM with a stable baseline.

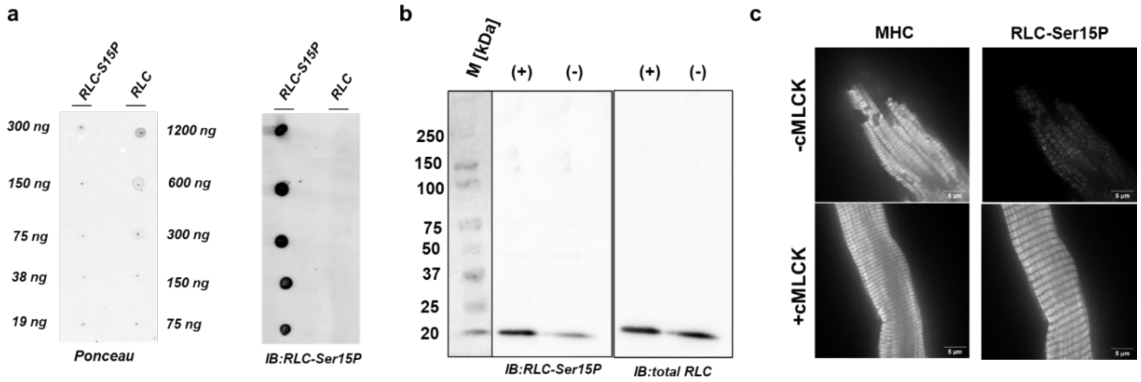

Fig. S3. Antibody validation. (a) Dot-blot of serine 15 phosphorylated (RLC-Ser15P) and unphosphorylated recombinant rat ventricular RLC using the anti-RLC-Ser15P antibody. Loaded mounts of recombinant protein are indicated accordingly. (b) Western-blot of myofibrils samples before (-) and after (+) cMLCK treatment using the anti-RLC-Ser15P antibody (left) and total RLC antibody (right). (c) Confocal images of myofibrils before (-MLCK) and after cMLCK (+cMLCK) treatment stained against myosin heavy chain (MHC) and serine 15 phosphorylated RLC.

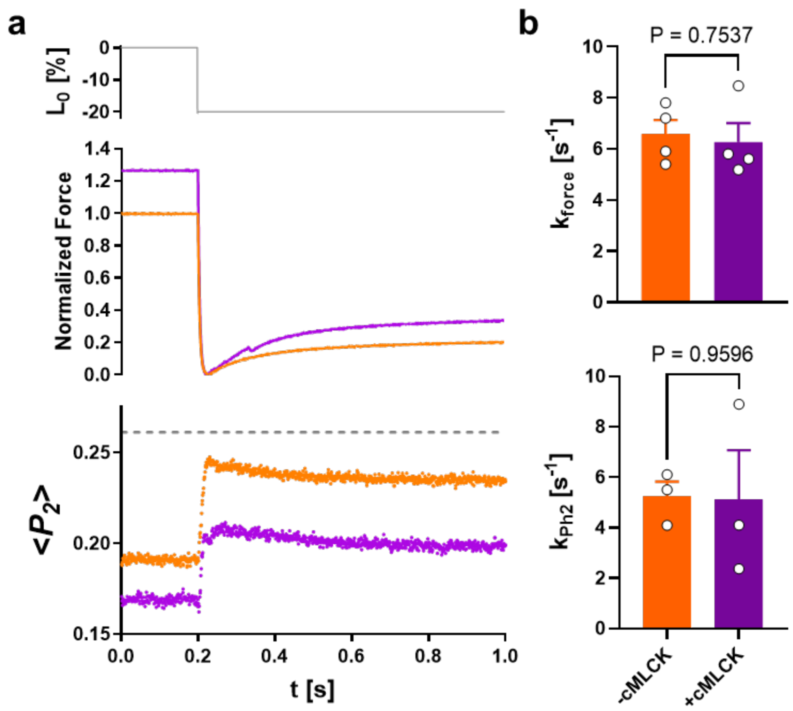

Fig. S4. Changes in the orientation of the cRLC E-helix probe in ventricular trabeculae in response to step shortening before and after cMLCK treatment. (a) Representative traces of muscle length (top), force (middle) and $<P_2>$ before (orange) and after RLC phosphorylation (purple). (b) Summary of k_{tr} (n=4 independent trabeculae preparations) and Ph2 rates (n=3 independent trabeculae preparations). Statistical significance of differences between values were assessed with a paired, two-tailed student's t-test.

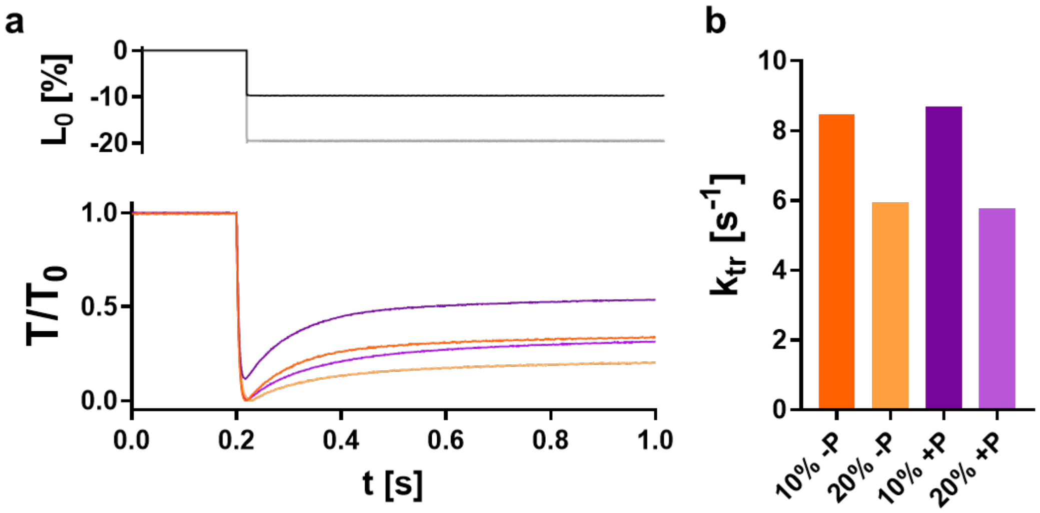

Fig. S5. Rate of force re-development of ventricular trabeculae after 10% and 20% shortening steps before (orange) and after RLC phosphorylation (purple). Representative traces are shown in (a) and data summarized in (b) for n=1 preparation.

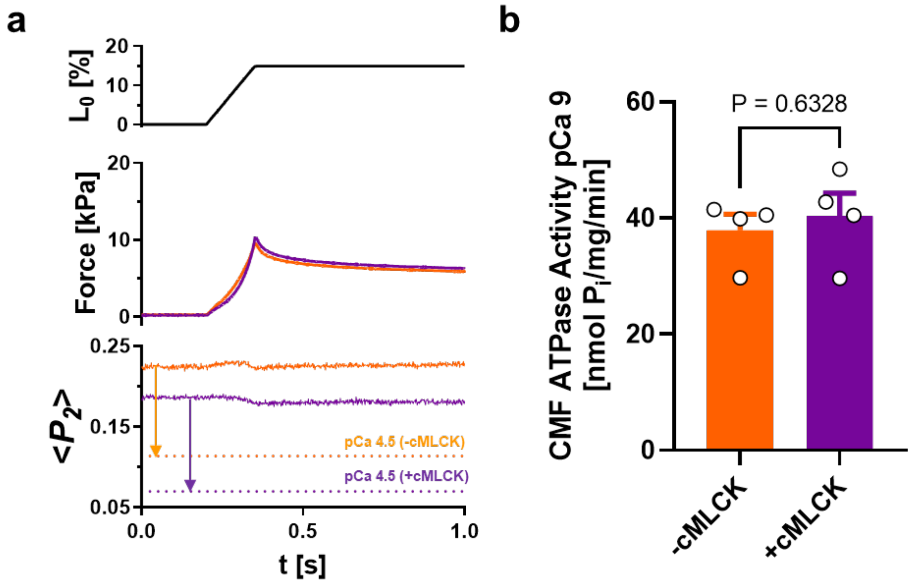

Fig. S6. Effect of RLC phosphorylation on (a) relaxed myosin head orientation in ventricular trabeculae 9 (n=1) and (b) ATPase activity of isolated myofibrils (n=4 independent preparations) at pCa 9. Statistical significance of differences between values were assessed with a paired, two-tailed students' t-test.
